# Pharmacological Profile of the Purinergic P2Y Receptors That Modulate, in Response to ADPβS, the Vasodepressor Sensory CGRPergic Outflow in Pithed Rats

**DOI:** 10.3390/ph16030475

**Published:** 2023-03-22

**Authors:** Alejandro D. Miguel-Martínez, Juan Linares-Bedolla, Belinda Villanueva-Castillo, Kristian A. Haanes, Antoinette MaassenVanDenBrink, Carlos M. Villalón

**Affiliations:** 1 Departamento de Farmacobiología, Cinvestav-Coapa, Czda. de los Tenorios 235, Col. Granjas Coapa, Deleg. Tlalpan, Ciudad de Mexico C.P. 14330, Mexico; alejandro.miguel@cinvestav.mx (A.D.M.-M.); juan.linares@cinvestav.mx (J.L.-B.); belyndavc@hotmail.com (B.V.-C.); 2 Department of Clinical Experimental Research, Glostrup Research Institute, Copenhagen University Hospital—Rigshospitalet, Nordstjernevej 42, DK-2600 Glostrup, Denmark; kristian.agmund.haanes@regionh.dk; 3 Division of Vascular Medicine and Pharmacology, Erasmus MC University Medical Center Rotterdam, P.O. Box 2040, 3000 CA Rotterdam, The Netherlands; a.vanharen-maassenvandenbrink@erasmusmc.nl

**Keywords:** ADPβS, glibenclamide, pithed rat, purinergic receptors, vasodepressor sensory CGRPergic tone

## Abstract

Calcitonin gene-related peptide (CGRP), an endogenous neuropeptide released from perivascular sensory nerves, exerts a powerful vasodilatation. Interestingly, adenosine triphosphate (ATP) stimulates the release of CGRP by activation of prejunctional P2X_2/3_ receptors, and adenosine 5′-O-2-thiodiphosphate (ADPβS), a stable adenosine diphosphate (ADP) analogue, produces vasodilator/vasodepressor responses by endothelial P2Y_1_ receptors. Since the role of ADP in the prejunctional modulation of the vasodepressor sensory CGRPergic drive and the receptors involved remain unknown, this study investigated whether ADPβS inhibits this CGRPergic drive. Accordingly, 132 male Wistar rats were pithed and subsequently divided into two sets. In set 1, ADPβS (5.6 and 10 µg/kg·min) inhibited the vasodepressor CGRPergic responses by electrical stimulation of the spinal T_9_–T_12_ segment. This inhibition by ADPβS (5.6 µg/kg·min) was reverted after i.v. administration of the purinergic antagonists MRS2500 (300 µg/kg; P2Y_1_) or MRS2211 (3000 µg/kg; P2Y_13_), but not by PSB0739 (300 µg/kg; P2Y_12_), MRS2211 (1000 µg/kg; P2Y_13_) or the K_ATP_ blocker glibenclamide (20 mg/kg). In set 2, ADPβS (5.6 µg/kg·min) failed to modify the vasodepressor responses to exogenous α-CGRP. These results suggest that ADPβS inhibits CGRP release in perivascular sensory nerves. This inhibition, apparently unrelated to activation of ATP-sensitive K^+^ channels, involves P2Y_1_ and probably P2Y_13_, but not P2Y_12_ receptors.

## 1. Introduction

Calcitonin gene-related peptide (CGRP) is a member of the endogenous peptides family, formed by 37 amino acids [1], which (i) was identified in plasma in the 1980s and subsequently in the spinal cord [2]; (ii) produces a potent vasodepressor effect, erythema and an increase in local blood flow [3,4]; and (iii) is mostly released by C sensory nerve fibres mainly emerging from dorsal root ganglia, trigeminal ganglia and heterogeneous small and medium-sized neurons [5,6,7]. Once released by sensory neurons, CGRP binds to the CGRP receptor, which is coupled to G_αs_ proteins and consists of two proteins, namely: (i) calcitonin-like receptor (CLR) and (ii) receptor activity modifying protein (RAMP_1_) [8,9].

The vasodilator actions of CGRP in rodents are mediated by indirect and direct vascular pathways that include (i) endothelium-dependent mechanisms associated with increases in cyclic adenosine monophosphate (cAMP), activation of protein kinase A (PKA), nitric oxide (NO) release and opening of adenosine triphosphate (ATP)-sensitive K^+^ channels (K_ATP_ channels) [10,11,12]; and (ii) musculotropic (vascular smooth muscle) mechanisms related to increases in cAMP, activation of PKA and opening of K_ATP_ channels [4,13,14]. Accordingly, stimulation of the vasodepressor sensory CGRPergic drive in normotensive and hypertensive rats results in a decrease in diastolic blood pressure (DBP) that involves both NO-dependent and NO-independent vasodilator pathways in peripheral resistance arteries [3,15,16,17,18,19,20].

CGRP release from sensory nerves can be prejunctionally stimulated by endogenous compounds such as anandamide [21], prostaglandins [22], bradykinin [22], acetylcholine [23] or ATP [24]. Moreover, our group has reported that in pithed rats (which have an inactive central nervous system) the vasodepressor sensory CGRPergic drive (which involves CGRP release from perivascular sensory nerves [3,4]) can be inhibited by prejunctional 5-HT_1B/1D_ [25,26,27], D_2_-like [28], α_2_-adrenergic [29] or H_3_ receptors [30]. Interestingly (i) ATP stimulates the CGRPergic drive by activating prejunctional P2X_2/3_ receptors which, in turn, results in systemic vasodilatation and a decrease in DBP [24,31,32,33,34]; (ii) adenosine diphosphate (ADP) is more potent than ATP and related compounds to produce vasodilatation and vasodepressor responses [35]; and (iii) adenosine 5′-O-2-thiodiphosphate (ADPβS; a stable and non-hydrolysable analogue of ADP) lowers DBP in anaesthetized rats [33], inhibits CGRP release from rat sensory neurons in dural arteries and trigeminal ganglion in situ by P2Y_13_ receptors [33], and inhibits the cardioaccelerator sympathetic drive in pithed rats mainly by P2Y_12_ receptors and less prominently by P2Y_13_ receptors [36].

Pharmacologically, ADPβS is an agonist with a preferential activity at purinergic P2Y_1_ (G_αq_-coupled), P2Y_12_ (G_αi/o_-coupled) and P2Y_13_ (G_αi/o_-coupled) receptors [37,38,39,40,41,42,43]. These receptors are widely expressed in the cardiovascular system, particularly modulating the function of endothelium, vascular smooth muscle, and autonomic nerves that innervate blood vessels and the heart [38,40,43,44,45,46,47,48]. Nevertheless, the role of P2Y_1_, P2Y_12_ and/or P2Y_13_ receptors in the modulation of the vasodepressor sensory CGRPergic drive is still unknown. Hence, in this pharmacological study in pithed rats we analysed the inhibition by ADPβS of the vasodepressor sensory CGRPergic drive by using the P2Y receptor antagonists MRS2500 (P2Y_1_), PSB0739 (P2Y_12_) and MRS2211 (P2Y_13_) [42,49,50], as well as the K_ATP_ channel blocker glibenclamide [10,11,12].

Our results suggest that ADPβS-induced inhibition of the vasodepressor sensory CGRPergic outflow, which seems to be unrelated to activation of ATP-sensitive K^+^ channels, could be mediated by activation of prejunctional P2Y_1_ and probably P2Y_13_, but not P2Y_12_, receptors.

## 2. Results

### 2.1. Systemic Haemodynamic Variables

The baseline values of DBP and heart rate after i.v. treatment with gallamine (25 mg/kg) followed by the continuous infusions of hexamethonium (2 mg/kg·min) and methoxamine (15–20 μg/kg·min) in the 132 pithed rats were 109 ± 9 mm Hg and 344 ± 3 beats/min. Table 1 shows that baseline DBP values (i) were significantly decreased (*p* < 0.05) 10 min after the i.v. continuous infusion of 5.6 or 10 µg/kg·min ADPβS had been started, and remained so during this infusion; and (ii) remained without significant changes (*p* > 0.05) 10 min after the i.v. infusions of 0.02 mL/min bidistilled water or 3 µg/kg·min ADPβS, or the i.v. bolus injections of 1 mL/kg bidistilled water, 300 µg/kg MRS2500, 300 µg/kg PSB0739, 1000 µg/kg MRS2211, 3000 µg/kg MRS2211, 1 mL/kg glibenclamide vehicle (33% PEG, 33% ethanol and 34% NaOH 0.2 M) or 20 mg/kg glibenclamide. The potential influence of this effect of ADPβS (or any other treatment) on the vasodepressor responses by electrical stimulation and exogenous α-CGRP was minimized by calculating these responses as a % change in DBP, as previously established [4,26,27,28,29,30]. Furthermore, it is to be noted that the decrease in DBP produced by ADPβS (5.6 µg/kg·min) (i) was blocked after i.v. administration of MRS2500 (300 µg/kg), glibenclamide vehicle (1 mL/kg) or glibenclamide (20 mg/kg); and (ii) remained unaltered after i.v. administration of MRS2211 (1000 µg/kg), MRS2211 (3000 µg/kg) or PSB0739 (300 µg/kg).

On the other hand, it must be emphasised that the baseline values of heart rate remained without changes (*p* > 0.05) after i.v. treatment with any of the above doses of compounds, as previously reported in pithed rats [36]. Hence, for the sake of clarity, these data are not shown.

### 2.2. Effect of Vehicle or ADPβS Infusions on the Vasodepressor Responses by Electrical Sensory Stimulation or Exogenous α-CGRP 

**Figure 1:**
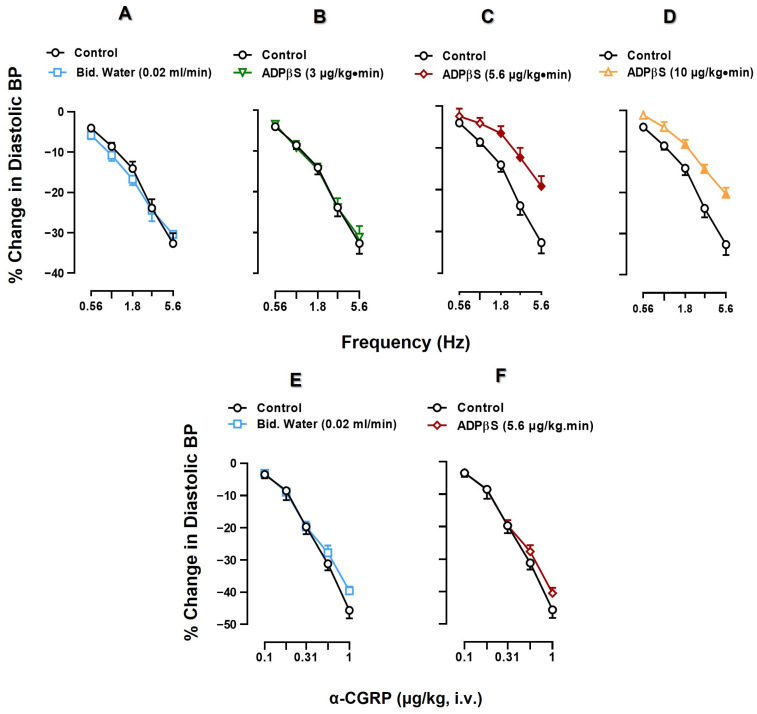
Comparative analysis of the effects produced by infusions of bidistilled water (Bid. Water) or ADPβS on the vasodepressor responses produced by electrical stimulation or exogenous α-CGRP. Effect of i.v. continuous infusions (after 10 min) of (**A**) bidistilled water (0.02 mL/min; ☐), (**B**) ADPβS (3 µg/kg·min; ▽), (**C**) ADPβS (5.6 µg/kg·min; ◊) and (**D**) ADPβS (10 µg/kg·min; △) on the vasodepressor CGRPergic responses (peak changes) induced by spinal electrical stimulation; and effect of i.v. continuous infusions (after 10 min) of (**E**) bidistilled water (0.02 mL/min; ☐) and (**F**) ADPβS (5.6 µg/kg·min; ◊) on the vasodepressor responses (peak changes) induced by i.v. bolus of exogenous α-calcitonin gene-related peptide (α-CGRP). Data are shown as percentage (%) change in diastolic blood pressure. Solid symbols indicate a significant difference (*p <* 0.05) against the corresponding control response (○). Values are indicated as means ± SEM *(n =* 6 for each subgroup).

Figure 1 compares the decreases in DBP produced by (i) electrical sensory stimulation (0.56–5.6 Hz; S-R curves, upper panel) and (ii) i.v. injections of α-CGRP (0.1–1 µg/kg; D-R curves, lower panel) in control animals (receiving no treatment), and in animals receiving infusions of bidistilled water (vehicle; 0.02 mL/min) or ADPβS (3 or 5.6 or 10 µg/kg·min).

Electrical sensory stimulation and the i.v. bolus administration of exogenous α-CGRP resulted in frequency-dependent (upper panel) and dose-dependent (lower panel) decreases in DBP represented as a percentage change in DBP. When comparing the vasodepressor responses of the control subgroup with those of the subgroup receiving vehicle (bidistilled water; 0.02 mL/min), no significant differences were found in the responses produced by sensory electrical stimulation or exogenous α-CGRP (*p >* 0.05) (Figure 1A,E). Thus, these vasodepressor responses were reproducible during our experimental protocols.

In contrast, the infusions of 5.6 and 10 µg/kg·min ADPβS produced a significant inhibition (compared to control) of the vasodepressor responses generated by sensory electrical stimulation at 1.8, 3.1 and 5.6 Hz (Figure 1C,D; *p <* 0.05), whereas 3 µg/kg·min ADPβS was inactive (Figure 1B; *p >* 0.05). However, as shown in Table 2, 5.6 and 10 µg/kg·min ADPβS produced practically the same degree of inhibition; in other words, this inhibition was not dose dependent. On this basis, the infusion dose of 5.6 µg/kg·min ADPβS was selected to investigate (i) its effect on the vasodepressor responses produced by exogenous α-CGRP; and (ii) the pharmacological profile of the P2Y receptors mediating ADPβS-induced inhibition of the vasodepressor sensory CGRPergic drive.

Indeed, Figure 1F indicates that 5.6 µg/kg·min ADPβS failed to inhibit the vasodepressor responses to exogenous α-CGRP (contrasting with Figure 1C). With these results, we suggest that the inhibition by ADPβS of the vasodepressor sensory CGRPergic drive is prejunctional in nature, but this does not shed further light on the specific pharmacological profile of the P2Y receptors involved. 

### 2.3. Effects of Vehicle, MRS2500, PSB0739 or MRS2211 on the Neurogenic Vasodepressor CGRPergic Responses by Electrical Stimulation 

**Figure 2:**
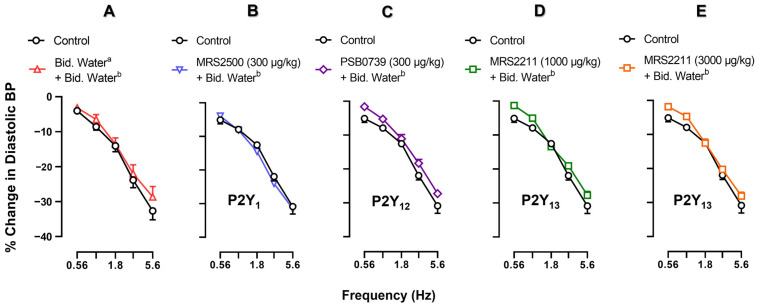
Comparative analysis of the effect of vehicle (bidistilled water) or P2Y receptor antagonists on the neurogenic vasodepressor responses produced by spinal (T_9_–T_12_) electrical stimulation. Effect of i.v. bolus injections (after 10 min) of (**A**) bidistilled water ^a^ (1 mL/kg; △), (**B**) MRS2500 (300 µg/kg; ▽), (**C**) PSB0739 (300 µg/kg; ◊), (**D**) MRS2211 (1000 µg/kg; ☐) or (**E**) MRS2211 (3000 µg/kg; ☐) on the decreases in DBP (peak changes) produced by electrical stimulation of the vasodepressor sensory CGRPergic drive during an i.v. continuous infusion (after 10 min) of bidistilled water ^b^ (0.02 mL/min). Empty symbols represent the control responses (○) or non-significant responses (☐, △, ▽, ◊) versus the control subgroup (*p >* 0.05). Values are indicated as means ± SEM *(n =* 6 for each subgroup).

One fundamental experimental condition that would facilitate the pharmacological analysis of ADPβS-induced inhibition of the neurogenic vasodepressor sensory CGRPergic responses is that these responses remain unaffected after the administration of a vehicle or P2Y receptor antagonists alone. For this purpose, Figure 2 compares the control vasodepressor CGRPergic responses by electrical stimulation (without treatment) with those produced after i.v. treatment with (A) bidistilled water (1 mL/kg; Figure 2A); (B) MRS2500 (300 μg/kg; P2Y_1_ antagonist, Figure 2B); (C) PSB0739 (300 μg/kg; P2Y_12_ antagonist, Figure 2C); (D) MRS2211 (1000 μg/kg; P2Y_13_ antagonist, Figure 2D); and (E) MRS2211 (3000 μg/kg; P2Y_13_ antagonist, Figure 2E).

Clearly, none of these pharmacological treatments produced a significant change on the S-R curves (*p >* 0.05). This means that, under our experimental conditions, these compounds have no effects on the neurogenic vasodepressor CGRPergic responses produced by electrical stimulation.

### 2.4. Effect of Vehicle, MRS2500, PSB0739 or MRS2211 on the ADPβS-Induced Inhibition of the Neurogenic Vasodepressor CGRPergic Responses by Electrical Stimulation

**Figure 3:**
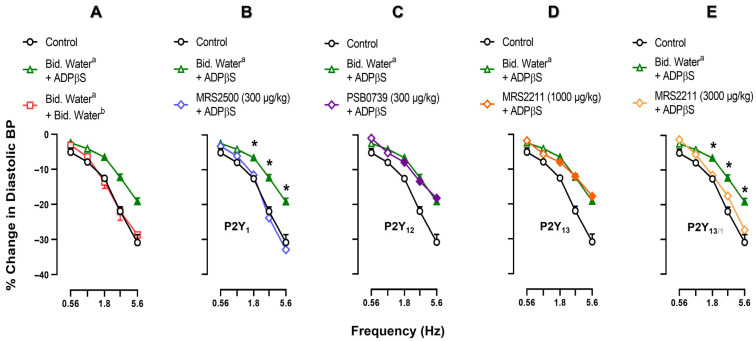
Effect of vehicles or P2Y receptor antagonists on the inhibition by ADPβS of the electrically-stimulated (T_9_–T_12_) vasodepressor sensory CGRPergic drive. Neurogenic vasodepressor responses (peak changes) produced by spinal electrical stimulation of the perivascular sensory CGRPergic nerves before (control) and following i.v. bolus injections (after 10 min) of (**A**) bidistilled water ^a^ (1 mL/kg) followed by an i.v. continuous infusion (after 10 min) of bidistilled water ^b^ (0.02 mL/min) or ADPβS (5.6 µg/kg·min); (**B**) MRS2500 (300 µg/kg), (**C**) PSB0739 (300 µg/kg), (**D**) MRS2211 (1000 µg/kg) or (**E**) MRS2211 (3000 µg/kg) followed by an i.v. continuous infusion (after 10 min) of ADPβS (5.6 µg/kg·min). Note that the results in panel A obtained from the subgroup receiving i.v. administration of bidistilled water ^a^ (1 mL/kg) + ADPβS (5.6 µg/kg·min) is also the same subgroup shown in panels B, C, D and E for comparative purposes with the subgroups receiving antagonists. Empty symbols represent the control responses (○) or nonsignificant responses (☐, △, ◊) versus the control responses (*p* > 0.05). Solid symbols indicate a significant difference (*p* < 0.05) versus the control responses (○). * Represents a significant difference (*p* < 0.05) versus the subgroup that received i.v. antagonist + ADPβS (5.6 µg/kg·min). Bid. Water stands for bidistilled water. Values are indicated as means ± SEM *(n =* 6 for each subgroup).

**Figure 4:**
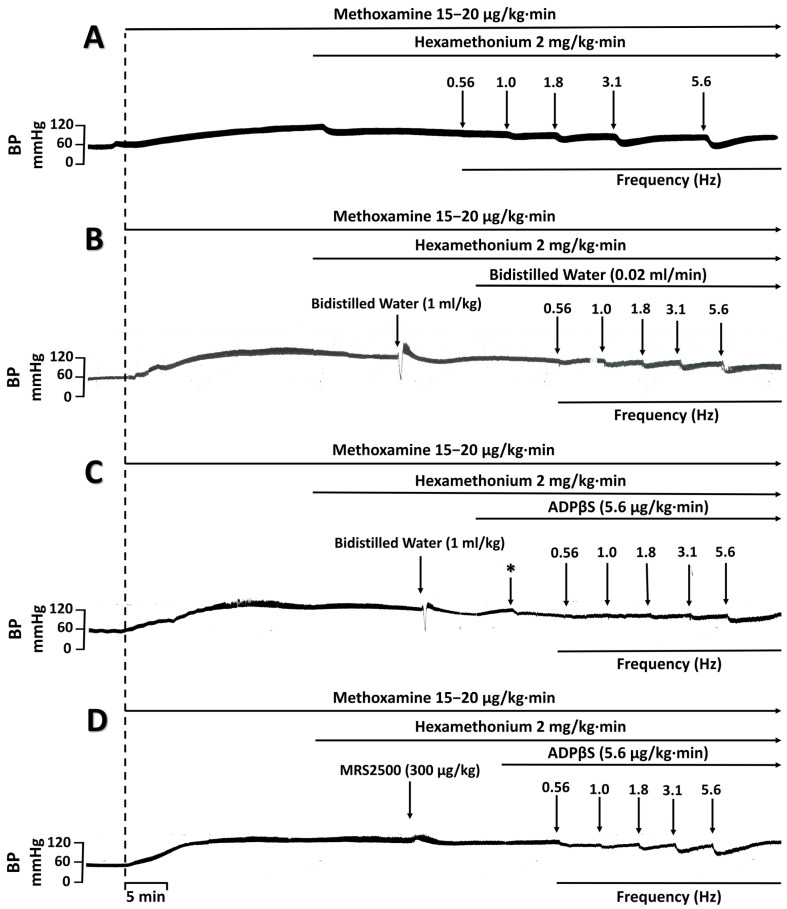
Experimental tracings of the vasodepressor responses by electrical stimulation of the vasodepressor sensory CGRPergic drive under different conditions. S-R curves of the vasodepressor responses (peak changes) by electrical stimulation of the perivascular sensory CGRPergic nerves in pithed rats with (**A**) no treatment (control); (**B**) i.v. bolus (after 10 min) of bidistilled water followed by i.v. infusions (after 10 min) of bidistilled water; (**C**) i.v. bolus (after 10 min) of bidistilled water followed by an i.v. infusion (after 10 min) of ADPβS (5.6 µg/kg·min); and (**D**) i.v. administration (after 10 min) of MRS2500 (300 µg/kg) followed by an infusion (after 10 min) of ADPβS (5.6 µg/kg·min). As shown in panel C, the ADPβS infusion (after 10 min) (i) produced a decrease in DBP (*, 19 ± 3 %; *p* < 0.05; Table 1); and (ii) inhibited the electrically-induced vasodepressor CGRPergic responses. Panel D also shows that these effects of ADPβS were blocked following i.v. administration (after 10 min) of MRS2500 (300 µg/kg). BP stands for blood pressure. Similar results were observed after each treatment (*n* = 6 for each subgroup).

Figure 3 illustrates the effects of an i.v. bolus injection of vehicle (bidistilled water; 1 mL/kg), MRS2500 (300 μg/kg; P2Y_1_ antagonist), PSB0739 (300 μg/kg; P2Y_12_ antagonist) or MRS2211 (1000 and 3000 μg/kg; P2Y_13_ antagonist) on the inhibition by ADPβS (5.6 µg/kg·min) of the neurogenic vasodepressor responses. The original experimental tracings of these results (excluding the effects of PSB0739 and MRS2211) are shown in Figure 4. Clearly, ADPβS-induced inhibition of the vasodepressor sensory CGRPergic drive remained unaffected after i.v. administration of (i) vehicle (1 mL/kg; *p* > 0.05 when comparing the responses shown in Figure 3A and Figure 1C) (see also Figure 4C); (ii) PSB0739 (300 μg/kg; Figure 3C) or MRS2211 (1000 μg/kg; Figure 3D). In contrast, ADPβS-induced inhibition was reverted after i.v. administration of MRS2500 (300 μg/kg; Figure 3B and Figure 4D) or MRS2211 (3000 μg/kg; Figure 3E). Interestingly, MRS2500 (300 μg/kg) also abolished the vasodepressor effect resulting from the infusion of ADPβS (Figure 4D and Table 1).

### 2.5. Effect of VEHICLE or glibenclamide on ADPβS-Induced inhibition of the Vasodepressor CGRPergic Responses by Electrical Stimulation

**Figure 5:**
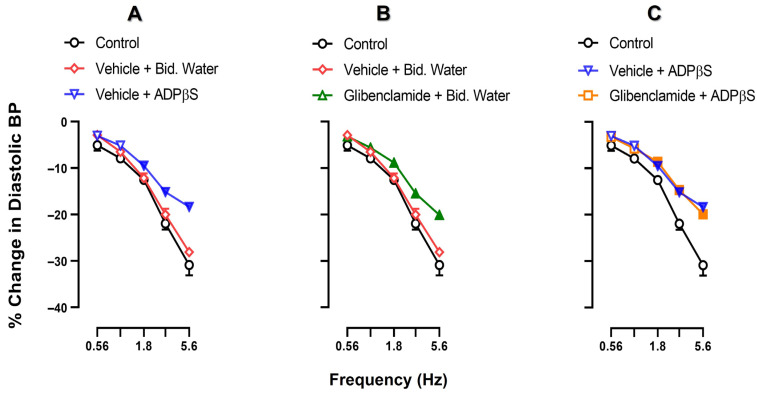
Comparative analysis of the effect of vehicle or glibenclamide on ADPβS-induced inhibition of the neurogenic vasodepressor CGRPergic responses. Effect on electrically-stimulated neurogenic vasodepressor CGRPergic responses (peak changes) of i.v. administration (after 10 min) of (**A**) a bolus of glibenclamide vehicle (1 mL/kg) followed by infusions (after 10 min) of bidistilled water (0.02 mL/min) or ADPβS (5.6 µg/kg·min); (**B**) an i.v. bolus (after 10 min) of glibenclamide (20 mg/kg) followed by an infusion (after 10 min) of bidistilled water (0.02 mL/min); and (**C**) an i.v. bolus (after 10 min) of glibenclamide (20 mg/kg) followed by an infusion (after 10 min) of ADPβS (5.6 µg/kg·min. Empty symbols represent the control responses (○) or nonsignificant responses (☐, △, ▽, ◊) versus the control subgroup (*p* > 0.05). Solid symbols indicate a significant difference (*p* < 0.05) against the control subgroup (○). Values are indicated as means ± SEM (*n* = 6 for each subgroup).

Figure 5 compares the control vasodepressor CGRPergic responses by electrical stimulation (without treatment) with those produced after an i.v. bolus injection of vehicle (1 mL/kg) or glibenclamide (20 mg/kg) followed by an i.v. continuous infusion of (i) bidistilled water (0.02 mL/min); or (ii) ADPβS (5.6 µg/kg·min). The i.v. administration of vehicle (1 mL/kg) followed by the infusion of bidistilled water (0.02 mL/min) produced no effect on the electrically-induced vasodepressor responses compared with the control (*p* > 0.05; Figure 5A), but the infusion of ADPβS (5.6 µg/kg·min) inhibited the electrically-induced vasodepressor responses (*p* < 0.05; Figure 5A). Interestingly, i.v. glibenclamide (20 mg/kg) followed by the infusion of bidistilled water (0.02 mL/min) significantly attenuated the electrically-induced vasodepressor responses (*p* < 0.05; Figure 5B) and did not revert ADPβS-induced inhibition (*p* > 0.05; Figure 5C).

## 3. Discussion

### 3.1. General

The pithed rat is a well-established experimental model for studying cardiovascular function [4,51] and has been further optimised for investigating the pharmacological profile of the receptors that modulate, at the peripheral level, the activity of the sympathetic and sensory CGRPergic nerve terminals that innervate the cardiovascular system [23,26,27,28,29,30,36,52,53,54,55,56,57]. Since central functions are not operative in this model, any change in blood pressure (and/or heart rate) produced by i.v. administration of any compound can be attributed exclusively to peripheral (rather than central) mechanisms [23,26,27,28,29,30,36,52,53,54,55,56,57]. On this basis, within the context of the present study, it is reasonable to assume that the inhibition of the vasodepressor sensory CGRPergic drive produced by ADPβS is peripheral in nature (i.e., at the level of perivascular sensory CGRPergic nerves, and unrelated to baroreceptor compensatory reflex mechanisms or central actions).

Hence, the present study has analysed the pharmacological profile of the purinergic P2Y receptors modulating the functionality of the CGRPergic neurovascular junction at the specific level of systemic resistance blood vessels, which are determinant for peripheral vascular tone and, consequently, for DBP. For this purpose, ADPβS (which is a preferential agonist at purinergic P2Y_1_, P2Y_12_ and P2Y_13_ receptors [33,36,39,47,58]) was used as it has recently been shown to produce (when given i.v.) (i) acute vasodepressor responses in anaesthetized rats [33]; and (ii) cardiac sympatho-inhibition in pithed rats by activation of purinergic P2Y_12_ receptors, and less prominently by P2Y_13_ receptors [36].

Our results show that ADPβS (5.6 µg/kg·min) is capable of producing a prejunctional inhibition of the vasodepressor sensory CGRPergic drive (implying an inhibition of CGRP release from perivascular sensory nerves) as it induced (i) inhibition of the vasodepressor responses produced by electrical stimulation of perivascular sensory CGRPergic nerves (Figure 1C); and (ii) no effect on the vasodepressor responses produced by exogenous α-CGRP (Figure 1F).

Moreover, it is to be noted that the electrically induced CGRP release from perivascular sensory nerves was not directly measured in our experiments but, alternatively, was determined by the evoked vasodepressor responses, as previously reported [26,27,28,29,30,54,55], which are specifically blocked by CGRP receptor antagonists [4,57].

### 3.2. Systemic Haemodynamic Variables

The sustained vasodepressor response produced by 5.6 and 10 µg/kg·min ADPβS (Table 1) is consistent with other studies reporting that (i) 1 and 10 µM ADPβS induce vascular smooth muscle hyperpolarization by the release of endothelium-derived hyperpolarising factor [47]; and (ii) 330 μg/kg ADPβS given i.v. in anaesthetised rats produces a biphasic blood pressure response consisting of an initial short-lasting vasodepressor response followed by a vasopressor response that was blocked after i.v. administration of 1000 µg/kg MRS2211 [33]. Thus, while the primary vasodepressor response to ADPβS (typically produced by low doses) could be mediated by endothelial P2Y_1_ receptors [37,38,45,46,47,48], the secondary vasopressor response to 330 μg/kg ADPβS is mediated by P2Y_13_ receptors [33]. 

The fact that the vasodepressor response produced by 5.6 µg/kg·min ADPβS was maintained after i.v. administration of PSB0739 (P2Y_12_ receptor antagonist) or MRS2211 (P2Y_13_ receptor antagonist) but was clearly blocked after MRS2500 (P2Y_1_ receptor antagonist) (Table 1), at doses that completely block their respective receptors in pithed rats [36], reinforces the involvement of P2Y_1_ receptors in this response. Indeed, P2Y_1_ receptors, which are expressed on endothelium and vascular smooth muscle, induce a plasma enhancement of [Ca^++^] via G_αq_ activation (GPCR), and this produces an increase in intracellular NO that results in vascular smooth muscle relaxation [35,37,38,44,46,47,48,50,59,60,61,62].

On the other hand, since glibenclamide blocked the response to ADPβS (Table 1), it would seem tempting to suggest the blockade of K_ATP_ channels a priori, given that these channels play a role in adenosine-induced vasodilatation [63]. Despite the fact that the glibenclamide vehicle also blocked this response (Table 1), we still considered it important to analyse the effect of glibenclamide on the ADPβS-induced inhibition of the vasodepressor sensory CGRPergic drive.

### 3.3. Effect of ADPβS on the Vasodepressor Sensory CGRPergic Drive

As shown in Figure 1 (upper panel), only 5.6 and 10 µg/kg·min ADPβS induced a significant inhibition of the vasodepressor CGRPergic responses by electrical stimulation at 1.8, 3.1, 5.6 Hz, but the degree of inhibition produced by the two infusions was practically identical (Table 2), probably producing a maximal (5.6 µg/kg·min) and a supramaximal (10 µg/kg·min) inhibition. Consequently, 5.6 µg/kg·min ADPβS was selected for the subsequent pharmacological analysis with exogenous α-CGRP and P2Y receptor antagonists. Hence, the fact that the vasodepressor responses to exogenous α-CGRP were not significantly modified (*p >* 0.05) by 5.6 µg/kg·min ADPβS (Figure 1, lower panel) suggests that ADPβS (i) inhibits the electrically-induced vasodepressor CGRPergic responses by activating prejunctional receptors; and (ii) does not interact with post-junctional (vascular musculotropic) receptors that might oppose CGRP-induced vasodilatation, for example, by activation of P2Y_1_ receptors producing vasoconstriction [42,50,59]. 

Evidence for a relationship between nucleotides and modulation of the vasodilator/vasodepressor CGRPergic tone was obtained from enhanced vasodepressor CGRPergic responses by pre-junctional purinergic receptors. In this regard, Holton (1959) reported that antidromic stimulation of a rabbit’s skin sensory nerves induced by the release of ATP, resulted in vasodepressor effects [32]. Subsequently, it was shown that the vasodepressor response to ATP, which activates heterodimeric purinergic P2X_2/3_ receptors on prejunctional sensory nerves, induces CGRP release [24,31,59]. Currently, only the interaction of ATP and adenosine on P2X and A1 receptor families, respectively, of pre-junctional sensory nerves has been described. Specifically, ATP released from sympathetic noradrenergic nerves interacts with P2X_2/3_ receptors on Aδ and C sensory nerves promoting the release of CGRP [31,32,35,40,49,50,55,58,59,60,62].

### 3.4. Inhibition of the Vasodepressor Sensory CGRPergic Drive by ADPβS: Possible Pharmacological Correlation with the Purinergic P2Y_1_, P2Y_12_ and P2Y_13_ Receptor Subtypes

Once the prejunctional inhibition by ADPβS of the vasodepressor sensory CGRPergic drive was established (Figure 1), our next step was to analyse the pharmacological profile of this response. For this purpose (as shown in Table 3), it is important to consider that (i) ADPβS can activate (and displays affinity for) P2Y_1_, P2Y_12_ and P2Y_13_ receptors [37,39,46,49]; and (ii) some antagonists for these receptors, which include MRS2500 (P2Y_1_), PSB0739 (P2Y_12_) and MRS2211 (P2Y_13_) [49], display specific binding affinities for these receptors.

It must be emphasised that the doses used of each of these antagonists were (i) based on their affinities for their respective receptor subtypes (Table 3); and (ii) sufficient to produce a complete blockade of their respective receptors in pithed rats [36] and in other experimental models [64,65,66,67,68,69,70,71,72,73]. In this respect, our pharmacological experience suggests that compounds with affinities (pK_i_ values) of 6, 7, 8 and 9 would require in vivo i.v. doses of approximately 3000, 1000, 300 and 100 µg/kg, respectively, to completely block their respective receptors. Hence, these lines of reasoning were considered for choosing the doses of compounds in the present study, namely: (i) 300 µg/kg MRS2500 (P2Y_1_); (ii) 300 µg/kg PSB0739 (P2Y_12_); and (iii) 1000 and 3000 µg/kg MRS2211 (P2Y_13_).

As a first step of our pharmacological investigation, we decided to explore the possible effects of each of these antagonists on the neurogenic vasodepressor CGRPergic responses produced by electrical stimulation. For this reason, they were administered during i.v. continuous infusions of the ADPβS vehicle (bidistilled water). Since no significant differences versus the control subgroup were found, this finding reinforces the view that these antagonists have no effects on baseline DBP (Table 1) and on the neurogenic vasodepressor CGRPergic responses (Figure 2). On the basis of their affinities shown in Table 3, their profile of blockade of cardiovascular responses in pithed rats [36], and the dosage considerations described above, the fact that the ADPβS-induced sensory inhibition was only reversed by i.v. MRS2500 (300 µg/kg) or MRS2211 (3000 µg/kg), but not by PSB0739 (300 µg/kg) or MRS2211 (1000 µg/kg) (Figure 3) suggests the main involvement of P2Y_1_, and probably P2Y_13_, but not P2Y_12_, receptors.

Clearly, the binding profile of MRS2211 as an antagonist for P2Y_13_ receptors (Table 3) is far from ideal and, certainly, does not guarantee selectivity in pithed rats. Thus, it could be argued that 3000 µg/kg MRS2211 reverted the inhibition by ADPβS because this high dose is capable of blocking P2Y_1_, P2Y_12_ and P2Y_13_ receptors, as reported earlier [64,65,66,67,68,69,70,71,72,73]. However, in our experiments (i) the doses used of MRS2500 and PSB0739 are high enough to selectively (Table 3) and completely block cardiovascular responses mediated by P2Y_1_ and P2Y_12_ receptors, respectively, in pithed rats [36]; and (ii) the inhibition by ADPβS was reverted by MRS2500 (300 µg/kg; Figure 3B), but not by PSB0739 (300 µg/kg; Figure 3C). Thus, the possibility exists that 3000 µg/kg MRS2211 could be blocking P2Y_1_ receptors. Notwithstanding, as shown in Table 1, the vasodepressor response resulting from 5.6 µg/kg·min ADPβS, which was abolished by MRS2500 as a typical response mediated by endothelial P2Y_1_ receptors [38,39,43,45,46,50], remained unaffected by MRS2211 (Table 1), as previously described by Haanes et al. [33]. Certainly, with the present results (Figure 3) we cannot exclude the possibility that in perivascular sensory CGRPergic nerves (i) the role of P2Y_13_ receptors is less prominent than that of P2Y_1_ receptors; and (ii) both P2Y_1_ and P2Y_13_ receptors are blocked by 3000 µg/kg MRS2211, but not by 1000 µg/kg MRS2211. Moreover, the vasodepressor response produced by 5.6 µg/kg·min ADPβS (mediated by endothelial P2Y_1_ receptors blocked by MRS2500; Table 1) might involve activation of endothelial NO synthase and promote vascular smooth muscle relaxation via activation of K_ATP_ channels by a cytosolic increase in Ca^++^ concentrations [38,39,43,45,46,50].

Our findings, suggesting the possible involvement of prejunctional P2Y_13_ receptors inhibiting CGRP release from perivascular sensory nerves (Figure 3E), are consistent with other studies reporting that ADPβS inhibits (i) CGRP release from rat sensory neurons in dural arteries and trigeminal ganglion by MRS2211-sensitive P2Y_13_ receptors [33]; and (ii) noradrenaline release from cardioaccelerator sympathetic nerves in pithed rats by activation of purinergic P2Y_12_ receptors and less prominently by P2Y_13_ receptors [36]. 

Regarding the possible transduction mechanisms of P2Y_13_ receptors associated with inhibition of neuronal CGRP release, some in vitro studies indicate that P2Y_13_ receptors have several transduction pathways [74,75,76] including, amongst others: (i) G_i/o_ protein activation with ADP, leading to inhibition of adenylate cyclase with a resulting decrease in cAMP production; (ii) phosphorylation of the PI3K/Akt/GSK3 axis that produces release of β-catenin and Nrf2 (transcription factors) promoting cell survival; and (iii) G_αq_ coupling, with a resulting increase in [Ca^++^] and activation of phospholipase C/PKC/ERK/CREB7DUSP2. Moreover, the βγ subunits can activate RhoA with a resulting decrease in Ca^++^ channel activity that modulates neurotransmitter release [74]. 

Our findings supporting the role of prejunctional P2Y_1_, and probably P2Y_13_, receptors in the inhibition of CGRP release from perivascular sensory nerves may complement the general concept of purinergic modulation of CGRP release in sensory neurons. With this concept in mind, activation of sympathetic postganglionic neurons results in the release of noradrenaline and ATP as a cotransmitter; in turn, ATP would activate P2X_2/3_ receptors (ATP-gated Na^+^, K^+^ and Ca^++^ channels [59,77]) on sensory nerves with an increase in CGRP release [24]. Subsequently, ATP at the neuroeffector junction would be hydrolysed to ADP by ecto-nucleoside triphosphate diphosphohydrolase (ecto-NTPDase 2,3,8) [78]; then ADP could stimulate P2Y_1_ and P2Y_13_ receptors on sensory neurons (as suggested in the present study) with a decrease in CGRP release.

### 3.5. Are K_ATP_ Channels Involved in the Inhibition of the Vasodepressor CGRPergic Drive by ADPβS?

K_ATP_ channels are expressed in vascular smooth muscle and modulate vascular tone, blood flow and blood pressure; when opened, they produce membrane hyperpolarization of vascular smooth muscle, relaxation and vasodilation [79,80,81,82]. Hence, glibenclamide (a K_ATP_ channel blocker) was used to pharmacologically discern the possible role of K_ATP_ channels in the CGRPergic sensory inhibition produced by ADPβS (Figure 5). However, under our experimental conditions, glibenclamide, which had no effect on DBP (Table 1), attenuated the vasodepressor sensory CGRPergic drive (Figure 5B), as previously reported [53]. Certainly, this effect could have overshadowed the ADPβS-induced sensory inhibition and would help explain why glibenclamide failed to revert ADPβS-induced inhibition of the vasodepressor sensory CGRPergic drive (compare Figure 5C with Figure 5A).

This inactivity of glibenclamide, notwithstanding, does not seem to be a compelling finding to rule out the role of K_ATP_ channels in ADPβS-induced prejunctional sensory-inhibition because we hypothesise that two fundamental mechanisms are operative in our experimental model, namely: (i) ADPβS-induced hyperpolarization of CGRPergic sensory nerves; and (ii) CGRP-induced systemic vasodilatation.

Within this context, on the one hand, the hyperpolarization of vascular smooth muscle cells resulting from activation by ADPβS of endothelial P2Y_1_ receptors [38,41,42,43,44,45,50,63] would produce activation of phospholipase C and, consecutively, an increase in cytoplasmic Ca^++^ concentrations, eNOS activity and release of NO which, in turn, would increase guanylate cyclase activity, phosphorylation of K_ATP_ channels, and K^+^ conductance leading to the relaxation of vascular smooth muscle [38,41,42,43,44,45,50,63].

On the other hand, the CGRP-induced systemic vasodilatation (via the activation of G_αs_ protein-coupled CGRP receptors) is mediated by two pathways: (i) direct smooth muscle vasorelaxation involving activation of adenylate cyclase and, consecutively, an increase in cAMP levels, PKA activity and phosphorylation of K_ATP_ channels; and (ii) endothelial vasorelaxation resulting from a sequential increase in PKA activity, NO production diffusing to vascular smooth muscle, guanylate cyclase activity, cGMP levels, and phosphorylation of K_ATP_ channels leading to vasodilation [10,13,14,18]. We would, finally, like to put forward (with no direct experimental evidence) that these transduction mechanisms activated by ADPβS at vascular level might also occur in perivascular sensory CGRPergic nerves.

### 3.6. Limitations of the Study

Based on the above, and considering the neurovascular junction, it is clear that glibenclamide may have blocked K_ATP_ channels at both prejunctional (perivascular sensory nerves) and postjunctional (vascular) levels and that, as a result, may have inhibited the actions of ADPβS and CGRP, respectively. For this reason, it was not possible to discern, under our experimental conditions, the actions of glibenclamide (blocking K_ATP_ channels) at prejunctional and postjunctional levels. These experimental limitations may be approached in other studies with additional technologies involving, among others, molecular biology and immunohistochemistry.

On the other hand, we have to recognize that (i) the comparison of affinities of agonists and antagonists at P2Y_1_, P2Y_12_ and P2Y_13_ receptors, shown in Table 3, consists of data obtained from human P2Y receptors; and (ii) as far as we know, this binding data comparison does not exist for rodents. Nevertheless, these binding data may be transferrable from humans to rodents for several reasons: (i) for ADPβS, the affinity is the same for human and rat P2Y_12_ receptors [65,83], but there are only limited differences between rat and human P2Y_13_ receptors [64]; (ii) for ADP, only comparable affinities exist for P2Y_1_ and P2Y_13_ receptors, which is equipotent on human and rat P2Y_1_ receptors [66,84], but it seems slightly more potent on human than on rat P2Y_13_ receptors [85]. The main finding of the present study is the blockade produced by MRS2500 on P2Y_1_ receptors (Figure 3B and Figure 4D), which displays a comparable affinity for human and rat P2Y_1_ receptors [86]. To our knowledge, rat binding data do not exist for both PSB0739 and MRS2211; however, for Ticagrelor (an FDA approved P2Y_12_ receptor antagonist), there was no difference in affinity for rodent and human P2Y_12_ receptors [87], suggesting a similar pharmacology.

### 3.7. Perspectives and Potential Clinical Significance

Purinergic P2Y receptors play an important role in numerous cardiovascular diseases including endothelial dysfunction, which is characterized by vasoconstriction, increased vascular permeability and a prothrombotic and proinflammatory state [44,46]. On the other hand, it has been suggested that CGRP is involved in cardiovascular pathologies such as hypertension [12,15,17,20,88] or neurovascular disorders such as migraines [6,33,89].

Based on the inactivity of PSB0739 (300 µg/kg; Figure 3C) to revert ADPβS-induced sensory inhibition, our results imply that, in healthy animals, there is no physiological relevance of purinergic P2Y_12_ receptors modulating CGRP release from perivascular sensory nerves. In keeping with this view, P2Y_12_ receptors are highly expressed on platelets and megakaryocytes, exerting a prothrombotic function. Nevertheless, in pathological conditions such as hypoxia, heart failure, hypertension, sepsis, atherosclerosis, tissue damage and inflammation (among others) P2Y_12/13_ receptors become relevant, generating (at an endothelial level) increased permeability, thrombosis and angiogenesis [46]. Significantly, the effectivity of the P2Y_1_ receptor antagonist MRS2500 (300 µg/kg; Figure 3B and Figure 4D) to revert ADPβS-induced inhibition strongly suggests that purinergic P2Y_1_ receptors may play a role in modulating the release of CGRP at a prejunctional level, in addition to their vasodilator effects. This would strengthen the role of P2Y_1_ receptors in vascular diseases such as hypertension and migraines.

## 4. Materials and Methods

### 4.1. Ethical Approval of the Study Protocol in Pithed Rats

As previously reported [26,27,28,29,30,36,54,55,56,57], “the experimental protocols of the present investigation were approved by our Institutional Ethics Committee on the use of animals on scientific experiments (CICUAL-Cinvestav; protocol number 0139-15), following the regulations stablished by the Mexican Official Norm (NOM-062-ZOO-1999) [90] in accordance with the guide for the Care and Use of Laboratory Animals in the USA [91], the ARRIVE guidelines for reporting experiments in animals [92] and the Legislation for the Protection of Animals Used for Scientific Purposes (Directive 2010/63/EU(2010)”. 

### 4.2. General Methods

A total of 132 male normotensive Wistar rats (380–420 g, 18–22 weeks of age) were used in the present investigation. The animals were maintained at 22 ± 2 °C room temperature, 50% humidity and a 12/12-h light/dark cycle (light beginning at 07:00 h) with food and water freely available in their home cages.

Following the methods described for stimulation of the vasodepressor sensory CGRPergic drive in pithed rats [26,27,28,29,30,54,55,57], “the rats were anaesthetized with sodium pentobarbital (60 mg/kg, i.p.); then, the animals were: (i) cannulated into the trachea and pithed by inserting a stainless steel rod through the ocular orbit and the foramen magnum into the vertebral foramen; and (ii) artificially ventilated with room air by using an Ugo Basile pump (56 strokes/min, stroke volume of 20 mL/kg; Ugo Basile Srl, Comerio, VA, Italy).

After bilateral cervical vagotomy, the rats were cannulated with polyethylene catheters which were placed in: (i) the left and right femoral veins for the continuous infusions of methoxamine and ADPβS (or vehicle), respectively; (ii) the left jugular vein for the continuous infusion of hexamethonium; and (iii) the right jugular vein, for the bolus injections of gallamine or the P2Y receptor antagonists (of vehicles). Subsequently, the left carotid artery was connected to a Grass pressure transducer (P23 XL), for the recording of blood pressure. Both, heart rate (measured with a 7P4F tachograph) and blood pressure were recorded simultaneously by a model 7D Grass polygraph (Grass Instrument Co., Quincy, MA, USA). The body temperature of each pithed rat (monitored with a rectal thermometer) was maintained at 37 °C by a lamp”.

### 4.3. Experimental Protocols

**Figure 6:**
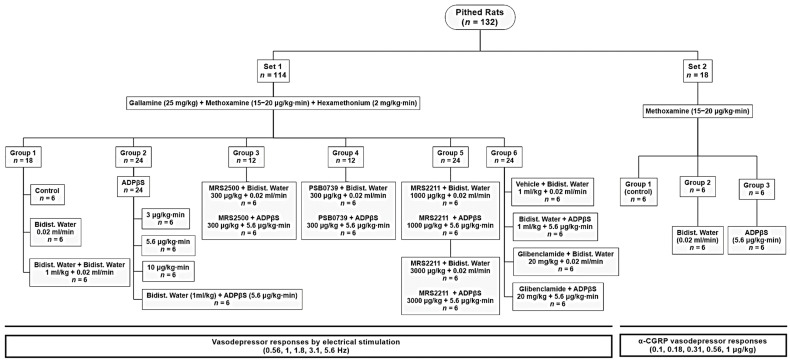
Experimental Design. Experimental protocols showing the number of pithed rats used in the two main sets and their subsequent division into different groups and subgroups.

Once the 132 pithed rats had been in a stable haemodynamic condition for at least 30 min, they were divided into two main sets for eliciting vasodepressor responses induced by (i) selective spinal (T_9_–T_12_) electrical stimulation of the vasodepressor sensory CGRPergic drive (set 1; *n* = 114), which represents the perivascular sensory CGRPergic nerves that innervate the systemic resistance blood vessels [26,27,28,29,30,54,55,57]; and (ii) i.v. bolus injections of exogenous α-CGRP (set 2; *n* = 18). The resulting vasodepressor stimulus-response curves (S-R curves; elicited by electrical stimulation) and dose-response curves (D-R curves; elicited by exogenous CGRP) were completed in about 50 min, with each electrical stimulus/dose given every 5–10 min, as established in previous studies [26,27,28,29,30,54,55,57]. As depicted in Figure 6, these 2 sets (corresponding to Protocols I and II; see below), in turn, were divided into different pre-treatment groups and, subsequently, into different subgroups (*n* = 6 each; see below). Then, the following experimental protocols followed.

#### 4.3.1. Protocol I: Selective Electrical Stimulation of the Vasodepressor Sensory CGRPergic Drive

In the first set (*n* = 114), “the stainless-steel rod was replaced by an enamelled electrode whose uncovered segment was located at T_9_–T_12_ of the spinal cord to allow selective stimulation of the vasodepressor sensory CGRPergic drive”, as previously reported [26,27,28,29,30,54,55,57]. Before electrical stimulation, the animals were pre-treated with gallamine (25 mg/kg, i.v.), a nondepolarizing neuromuscular blocking agent, to avoid the electrically induced muscular twitching [25,36,56]. In order to obtain vasodepressor responses, “DBP was initially increased and maintained at around 100–120 mm Hg by an i.v. continuous infusion of methoxamine (15–20 μg/kg·min) during and until the end of the experiments”, as previously established by our group [26,27,28,29,30,54,55,57]. Then, the animals received i.v. continuous infusions of hexamethonium (2 mg/kg·min), a nicotinic ganglion blocker, to block the sympathetic vasopressor responses generated by electrical stimulation of the spinal T_9_ segment [4,25,56,57]. When haemodynamic conditions were stable, baseline values of heart rate and DBP (a more accurate indicator of peripheral vascular resistance) were determined [36], and the 114 animals were then divided into six groups (*n* = 18, 24, 12, 12, 24, 24, respectively) for spinal T_9_–T_12_ electrical stimulation (see Figure 6). It must be emphasised that, prior to electrical spinal stimulation (and also prior to i.v. bolus injections of exogenous α-CGRP) to produce vasodepressor responses (depending on the specific protocol for each subgroup; see below), 10 min were allowed to elapse after each i.v. bolus injection of compound and after each i.v. continuous infusion of compound.

Spinal T_9_–T_12_ electrical stimulation consisted of applying trains of 10 s to selectively stimulate the vasodepressor sensory CGRPergic drive (monophasic rectangular pulses of 2 ms and 50 V) at increasing frequencies (0.56, 1.0, 1.8, 3.1 and 5.6 Hz). When DBP had returned to baseline levels, the next frequency was applied (at intervals of about 5–10 min) until the S-R curve was completed (around 50 min).

The first group (*n* =18) was subdivided into three subgroups (*n* = 6 each) that received (i) no pharmacological treatment (control subgroup); (ii) an i.v. continuous infusion of bidistilled water (0.02 mL/min); and (iii) an i.v. bolus injection of bidistilled water (1 mL/kg) followed by an i.v. continuous infusion of bidistilled water (0.02 mL/min).

The second group (*n* = 24) was subdivided into four subgroups (*n* = 6 each) to determine the effect of increasing infusion doses of ADPβS, namely: (i) ADPβS (3 μg/kg·min); (ii) ADPβS (5.6 μg/kg·min); (iii) ADβS (10 μg/kg·min); and (iv) an i.v. bolus of bidistilled water (1 mL/kg) followed by ADPβS (5.6 μg/kg·min). 

The third group (*n* = 12) was subdivided into two subgroups (*n* = 6 each) to evaluate the effect of an i.v. bolus injection of the P2Y_1_ receptor antagonist: (i) MRS2500 (300 µg/kg) followed by an i.v. continuous infusion of bidistilled water (0.02 mL/min); and (ii) MRS2500 (300 µg/kg) followed by an i.v. continuous infusion of ADPβS (5.6 μg/kg·min). 

The fourth group (*n* = 12) was subdivided into two subgroups (*n* = 6 each) to investigate the effect of an i.v. bolus injection of the P2Y_12_ receptor antagonist: (i) PSB0739 (300 µg/kg) followed by an i.v. continuous infusion of bidistilled water (0.02 mL/min); and (ii) PSB0739 (300 µg/kg) followed by an i.v. continuous infusion of ADPβS (5.6 μg/kg·min). 

The fifth group (*n* = 24) was subdivided into four subgroups (*n* = 6 each) to analyse the effect of an i.v. bolus injection of the P2Y_13_ receptor antagonist: (i) MRS2211 (1000 μg/kg) followed by an i.v. continuous infusion of bidistilled water (0.02 mL/min); (ii) MRS2211 (1000 μg/kg) followed by an i.v. continuous infusion of ADPβS. (5.6 μg/kg·min); (iii) MRS2211 (3000 μg/kg) followed by an i.v. continuous infusion of bidistilled water (0.02 mL/min); and (iv) MRS2211 (3000 µg/kg) followed by an i.v. continuous an i.v. infusion of ADPβS (5.6 μg/kg·min).

The sixth group (*n* = 24) was divided into four subgroups (*n* = 6) to explore the role of K_ATP_ channels. For this purpose, these subgroups received, individually, an i.v. bolus of (i) vehicle (1 mL/kg) followed by an i.v. continuous infusion of bidistilled water (0.02 mL/min); (ii) vehicle (1 mL/kg) followed by an i.v. continuous infusion of ADPβS (5.6 μg/kg·min); (iii) glibenclamide (20 mg/kg) followed by an i.v. continuous infusions of bidistilled water (0.02 mL/min); and (iv) glibenclamide (20 mg/kg) followed by an i.v. continuous infusion of ADPβS (5.6 μg/kg·min).

The doses of the above antagonists/blockers have been shown to abolish the responses mediated by their corresponding receptors/mechanisms in pithed rats [36,53].

#### 4.3.2. Protocol II: Intravenous Bolus Injections of Exogenous α-CGRP

The second set of pithed rats (*n* = 18) was prepared as described above, but the pithing rod was left throughout the experiment, and the administration of both gallamine and hexamethonium was omitted, as previously established [26,27,28,29,30,54,55,57]. Once the animals maintained a stable haemodynamic condition for 30 min, baseline DBP values were determined, and the animals were divided into three subgroups (*n* = 6 each) that were given (i) no pharmacological treatment (control subgroup); (ii) an i.v. continuous infusion of bidistilled water (0.02 mL/min); and (iii) an i.v. continuous infusion of ADPβS (5.6 μg/kg·min).

“Ten min later, these subgroups received consecutive i.v. bolus injections of exogenous α-CGRP at increasing doses (0.1, 0.18, 0.31, 0.56, and 1 μg/kg) to produce dose-dependent vasodepressor responses. When DBP returned to baseline levels, the next dose was applied (about 5 min) until the D-R curve was completed (approximately 30 min)”, as reported earlier [26,27,28,29,30,54,55,57].

### 4.4. Supplementary Procedures

It is to be noted that the doses of (i) vehicle (bidistilled water) or ADPβS were continuously infused (i.v.) at a rate of 0.02 mL/min by a KDS100 model infusion pump (KD Scientific Inc., Holliston, MA, USA); and (ii) vehicles or antagonists were given as i.v. bolus injections in volumes of 1 mL/kg.

The intervals between the different stimulation frequencies or α-CGRP doses depended on the duration of the vasodepressor responses (5 min), as we waited until DBP had returned to baseline values.

Moreover, since the CGRPergic vasodepressor responses (produced by electrical stimulation or exogenous α-CGRP) are highly tachyphylactic [4,26,27,28,29,30,54,55,57] (unlike the vasopressor responses by sympathetic stimulation or exogenous noradrenaline [25,56]), we decided not to perform more than one S-R or D-R curve (Figure 6).

### 4.5. Compounds

During the development of these experimental protocols, the compounds employed in the present study (obtained from the sources indicated) were sodium pentobarbital (PISA Agropecuaria, Mexico City, Mexico); gallamine triethiodide, hexamethonium chloride, glibenclamide, rat α-CGRP, methoxamine hydrochloride and adenosine-5′-[β-thio]diphosphate trilithium salt (ADPβS) (Sigma Chemical Co., St. Louis, MO, USA); (1R*,2S*)-4-[2-Iodo-6-(methylamino)-9H-purin-9-yl]-2-(phosphonooxy) bicyclo [3.1.0]hexane-1-methanol dihydrogen phosphate ester tetra ammonium salt (MRS2500); 1-amino-9,10-dihydro-9,10-dioxo-4-[[4-(phenylamino)-3-sulfophenyl]amino]-2-anthracenesulfonic acid sodium salt (PSB0739) and 2-[(2-chloro-5-nitrophenyl)azo]-5-hydroxy-6-methyl-3-[(phosphonooxy)methyl]–4-pyridinecarboxaldehyde disodium salt (MRS2211) (TOCRIS, Avonmouth, Bristol, UK).

As previously reported (i) “gallamine, hexamethonium, α-CGRP and methoxamine were dissolved in physiological saline” [26,27,28,29,30,54,55,57]; (ii) “ADPβS, MRS2500, PSB0739 and MRRS2211 were dissolved in bidistilled water” [36]; and (iii) “glibenclamide was dissolved in a vehicle combination of 33% PEG, 33% ethanol and 34% NaOH 0.2 M” [53]. None of these vehicles affected the baseline values of DBP or heart rate (not shown).

### 4.6. Data Presentation and Statistical Evaluation

“All data in the text and figures are presented as the mean ± SEM. The peak changes in DBP by electrical stimulation or exogenous α-CGRP were expressed as the percent change from baseline”, as previously described in pithed rats [26,27,28,29,30,54,55,57]. “A one-way ANOVA was used to compare the absolute values of DBP obtained during the continuous infusions of methoxamine, before and 10 min after the administration of all compounds before starting the electrical stimulation.

Moreover, the decreases in DBP induced electrically or by exogenous α-CGRP in the different subgroups of animals were evaluated with the Student-Newman-Keuls post hoc test, once a two-way repeated measures ANOVA (randomized block design) showed that the samples represented different populations [93]”, as reported in previous studies [26,27,28,29,30,54,55,57]. Statistical significance was accepted at *p* < 0.05. Statistical analysis was performed using SigmaPlot 12.0 (Systat Software, Inc. SigmaPlot for Windows).

The graphics were performed using Prism 6 software (GraphPad Software, Inc., San Diego, CA, USA).

## 5. Conclusions

Our results, taken together, allow us to suggest that the inhibition of vasodepressor sensory CGRPergic outflow produced by 5.6 mg/kg·min of ADPβS in healthy pithed rats (i) is apparently unrelated to activation of ATP-sensitive K^+^ channels; and (ii) could be mediated by activation of prejunctional P2Y_1_ and probably P2Y_13_, but not P2Y_12_, receptors.

## Figures and Tables

**Table 1:** Values of diastolic blood pressure (DBP) in pithed rats after i.v. treatment with gallamine followed by the continuous infusions of hexamethonium and methoxamine before (baseline) and 10 min after i.v. administration of several compounds.

Treatment	Doses	DBP (mm Hg)		
		Before	After 10 min	% Change DBP
Control (no treatment)		108 ± 4	−	0 ± 0
Bidistilled water	1 mL/kg ^a^	108 ± 4	113 ± 4	4 ± 3
Bidistilled water	0.02 mL/min ^b^	103 ± 2	103 ± 3	1 ± 1
ADPβS (adenosine 5′-O-2- thiodiphosphate)	3 µg/kg·min ^b^	116 ± 5	110 ± 5	−5 ± 2
	5.6 µg/kg·min ^b^	111 ± 3	90 ± 5 ^Δ^	−19 ± 3 ^Δ◊□^
	10 µg/kg·min ^b^	105 ± 3	73 ± 3 ^Δ^	−30 ± 5 ^Δ◊□○^
MRS2500	300 µg/kg ^a^	110 ± 3	111 ± 3	2 ± 3
MRS2500 + ADPβS	300 µg/kg ^a^ + 5.6 µg/kg·min ^b^	114 ± 6	114 ± 5	0 ± 1
PSB0739	300 µg/kg ^a^	106 ± 4	109 ± 4	2 ± 2
PSB0739 + ADPβS	300 µg/kg ^a^ + 5.6 µg/kg·min ^b^	116 ± 4	97 ± 5	−17 ± 2 ^Δ◊□^
MRS2211	1000 µg/kga	109 + 3	109 ± 3	0 ± 0
MRS2211 + ADPβS	1000 µg/kga + 5.6 µg/kg·minb	114 ± 4	94 ± 5	−18 ± 3 ^Δ◊□^
MRS2211	3000 µg/kga	110 ± 2	110 ± 2	0 ± 0
MRS2211 + ADPβS	3000 µg/kg ^a^ + 5.6 µg/kg·min ^b^	120 ± 3	103 ± 3	−13 ± 2 ^Δ◊□^
Vehicle of glibenclamide	1 mL/kg ^a^	107 ± 4	106 ± 5	−1 ± 2
Vehicle of glibenclamide + ADPβS	1 mL/kg ^a^ + 5.6 µg/kg·min ^b^	106 ± 3	106 ± 3	0 ± 0
Glibenclamide	20 mg/kg ^a^	108 ± 2	108 ± 2	0 ± 0
Glibenclamide + ADPβS	20 mg/kg ^a^ + 5.6 µg/kg·min ^b^	103 ± 1	102 ± 2	−1 ± 2

Doses were given as ^a^ i.v. bolus injections or ^b^ i.v. continuous infusions. (^Δ^) *p* < 0.05 versus control; (^◊^) *p* < 0.05 versus continuous infusions of vehicle; (^□^) *p* < 0.05 versus adenosine 5′-O-2-thiodiphosphate (ADPβS [3 µg/kg·min]); (^○^) *p* < 0.05 versus ADPβS (5.6 µg/kg·min). Values are indicated as means ± SEM (*n* = 6 for each subgroup).

**Table 2:** Percentage of the inhibition produced by the infusions (after 10 min) of vehicle (bidistilled water; 0.02 mL/min) or ADPβS (3, 5.6 and 10 µg/kg·min) on the vasodepressor CGRPergic responses (peak changes) by electrical sensory stimulation in pithed rats.

Group	0.56 Hz	1.0 Hz	1.8 Hz	3.1 Hz	5.6 Hz
Control	−4.0 ± 0.3	−8.5 ± 0.9	−14.0 ± 1.6	−23.8 ± 2.1	−32.6 ± 2.5
Vehicle (0.02 mL/min)	−4.9 ± 1.3	−10.7 ± 1.3	−16.7 ± 1.4	−24.4 ± 2.7	−30.4 ± 2.4
ADPβS (3 µg/kg·min)	−3.5 ± 0.4	−9.2 ± 1.7	−14.5 ± 1.5	−23.9 ± 2.3	−31.2 ± 2.8
ADPβS (5.6 µg/kg·min)	−2.4 ± 0.7	−4.1 ± 0.5	−6.4 ± 0.6 ^Δ◊□^	−12.2 ± 0.9 ^Δ◊□^	−19.1 ± 1.0 ^Δ◊□^
ADPβS (10 µg/kg·min)	−1.1 ± 1.1	−4.1 ± 1.3	−8.2 ± 1.1 ^Δ◊□^	−14.3 ± 1.2 ^Δ◊□^	−20.4 ± 1.2 ^Δ◊□^

(^Δ^) *p <* 0.05 versus control; (^◊^) *p <* 0.05 versus continuous infusions of bidistilled water; (^□^) *p <* 0.05 versus ADPβS (3 µg/kg·min); values are indicated as means ± SEM (*n =* 6 for each subgroup).

**Table 3:** Affinity values of some P2Y ligands for P2Y_1_, P2Y_12_ and P2Y_13_ receptors.

	Receptors	P2Y_1_	P2Y_12_	P2Y_13_
Drugs				
Agonist	ADPβS	5.6	7.5	7.5
Antagonists	MRS2500	9.1	4 ^a^	4 ^a^
	PSB0739	6 ^b^	7.6	6 ^b^
	MRS2211	5 ^c^	5 ^c^	6.3

Values are presented as *p*K_i_/*p*EC_50_/*p*IC_50_ in human receptors. No effect was observed until ^a^ 100 μM; ^b^ 1 μM; ^c^ 10 μM. Data taken and modified from [36,37].

## Data Availability

Not applicable.

## Abbreviations

ADP	Adenosine diphosphate
ADPβS	Adenosine 5′-O-2-thiodiphosphate
ATP	Adenosine triphosphate
cAMP	Cyclic adenosine monophosphate
cGMP	Cyclic guanosine monophosphate
CGRP	Calcitonin gene-related peptide
CGRPR	Calcitonin gene-related peptide receptor
CLR	Calcitonin-like receptor
DBP	Diastolic blood pressure
D-R curves	Dose-response curves
Ecto-NTPDase 2,3,8	Ecto-nucleoside triphosphate diphosphohydrolase
eNOS	Endothelial nitric oxide synthase
GPCR	G protein-coupled receptor
NO	Nitric oxide
PKA	Protein kinase A
RAMP_1_	Receptor activity modifying protein
S-R curves	Stimulus-response curves
